# The Impact of Urinary Tract Infections in Kidney Transplant Recipients: A Six-Year Single-Center Experience

**DOI:** 10.7759/cureus.44458

**Published:** 2023-08-31

**Authors:** Abdulrahman R Al Tamimi, Wed S Alotaibi, Renad M Aljohani, Sarah S Aldharman, Noof M Alharbi, Haneen S Khair

**Affiliations:** 1 Hepatobiliary Sciences and Organ Transplantation, King Abdulaziz Medical City, Riyadh, SAU; 2 College of Medicine, King Saud Bin Abdulaziz University for Health Sciences, Riyadh, SAU; 3 Research, King Abdullah International Medical Research Center, Riyadh, SAU

**Keywords:** infection, antibiotics, bacteriuria, kidney transplantation, urinary tract infection

## Abstract

Background

Urinary tract infections (UTIs) are the most common infections following kidney transplantation. Risk factors for UTIs in kidney transplant recipients include female gender, age, pre-transplant urinary tract abnormalities, diabetes, and recurrent UTIs. Infections of the bladder or urethra are termed lower UTIs, while those involving the kidneys or ureters are called upper UTIs.

Methods

We retrospectively screened our hospital information system to identify all patients who underwent kidney transplantation in the surgery department at King Abdulaziz Medical Center in Riyadh. A total of 553 records were ultimately included in the final analysis.

Results

A total of 553 patients were ultimately included in the analysis. More than half of the patients were males (62.4%), and 31.1% were aged between 45 and 60 years. Of these, 230 patients had a UTI, representing 41.59% of the sample. The most commonly reported causes were hypertensive nephrosclerosis (43.4%) and diabetic nephropathy (28.8%). The most frequently isolated causative agents were *Escherichia coli *(51.0%) and *Klebsiella pneumoniae* (21.8%). UTIs were significantly more prevalent among females, accounting for 73.6% of cases. Antibiotics were used in 86.5% of patients, with trimethoprim/sulfamethoxazole (65.8%) and ciprofloxacin (54.8%) being the most commonly prescribed.

Conclusion

About half of the patients in this study suffered from UTIs. *E. coli* and *K. pneumoniae *were the most commonly isolated organisms. Trimethoprim/sulfamethoxazole and ciprofloxacin were the most frequently prescribed antibiotics. A significant association was found between acquiring a UTI and being female (p < 0.001), as well as having pre-existing bladder dysfunction (p = 0.024).

## Introduction

Urinary tract infections (UTIs) that occur early after surgery are the most common infections after kidney transplantation (KT) [1]. According to the Centers for Disease Control and Prevention (CDC), asymptomatic bacteriuria is defined as a positive urine culture (identified as >105 colony-forming units (CFU)) with no associated symptoms of UTI and no systemic clinical symptoms. Uncomplicated acute simple cystitis is defined as a positive urine culture (identified as >105 CFU) associated with local urinary symptoms such as dysuria and frequency, but with no systemic symptoms or flank pain. Complicated UTI is characterized by a positive urine culture (identified as >105 CFU) with systemic manifestations such as fever and one of the following clinical signs: flank pain, costovertebral angle tenderness, or biopsy findings consistent with pyelonephritis. Recurrent UTI is defined as two episodes of acute bacterial cystitis with associated symptoms within the last six months or three episodes within the last year [2].

Risk factors for UTIs in kidney transplant recipients are similar to those in the general population and include female gender, age, pre-transplant urinary tract abnormalities, diabetes, and recurrent UTI [3]. Furthermore, urinary tract infections are caused by microorganisms, usually bacteria, that invade the urethra and bladder, leading to inflammation and infection. UTIs most commonly affect the urethra and bladder, but bacteria can also travel up the ureter to infect the kidneys. Patients with KT are exposed to a broader spectrum of pathogens than those without KT due to prior antibiotic treatments, hospitalizations, and urinary tract catheterizations. Additionally, UTIs after KT are usually caused by Gram-negative bacteria, with Escherichia coli being the most common pathogen, accounting for 30-80% of cases [4]. Other Gram-negative bacteria, such as *Klebsiella*, *Proteus*, and *Pseudomonas aeruginosa*, are also frequently isolated. Meanwhile, Gram-positive pathogens like *Streptococcus *species and *Staphylococcus saprophyticus *are less common [4].

Globally, one Turkish study investigated the risk factors for urinary tract infections in kidney transplant patients and their effects on graft function [5]. A total of 145 patients were included in the study, 66.2% of whom were men. During the first year following the transplant, 37.9% of the patients had 105 episodes of urinary tract infections. Female sex, glomerulonephritis as a primary kidney disease, pre-transplant diabetes, and the presence of a ureteral stent were all found to be risk factors for urinary tract infections. *Escherichia coli *and *Klebsiella pneumoniae *were the most common pathogens identified. The mean glomerular filtration rate at 12 months in patients with a urinary tract infection was significantly lower than in patients without an infection [5].

A retrospective cohort study of 380 recipients who developed symptomatic UTI within one year of transplantation was conducted [6]. During the first year after a kidney transplant, approximately 48.4% of patients acquired symptomatic UTIs; 21.8% of them developed early UTIs, about 13.2% developed late UTIs, and 13.4% had recurrent UTIs. All patients showed significant improvement in graft function after three years except for those with recurrent urinary tract infections, who had a poorer glomerular filtration rate (eGFR 60 mL/min/1.73 m^2^). Therefore, symptomatic UTIs that recur in the first year after transplantation have a long-term adverse effect on graft function [6].

Moreover, another study was conducted to investigate the impact of severe UTIs after renal transplantation on both graft function and healthcare resources [7]. They performed a retrospective review of kidney transplant recipients (KTRs) who had severe UTIs that necessitated hospitalization. They found 198 UTI-related hospitalizations among 83 kidney transplant recipients, which represents 7.4% of transplant-related hospitalizations; 44.6% of them were men. *E. coli *(47.5%) and *Klebsiella *(16.2%) were the most frequently isolated pathogens [7].

Locally, a study was conducted at the Hamed Al-Essa Organ Transplant Center in Kuwait to evaluate risk factors for recurrent UTIs and their long-term outcomes in kidney transplant recipients. The study included 1,019 patients who underwent kidney transplants between 2000 and 2012. Factors such as age, gender, pre-transplant urinary tract abnormalities, retransplantation, mode of induction and maintenance of immunosuppression, and associated comorbidities were identified as risk factors. The study particularly focused on recipients who experienced recurrent UTIs, defined as four or more episodes a year or two or more every six months. Graft function levels and timing of graft outcomes were compared using serum creatinine levels after one year, and no significant difference was found (P > .05). It was observed that recurrent UTI was most common among women and increased the risk of vaginal colonization by uropathogens [8].

Another retrospective chart review was conducted at King Abdulaziz Medical City and included 279 renal transplant recipients. Of these, 35% experienced a UTI within the first six months following the transplant, with the majority occurring in the second month. The recurrence of UTI was also most frequent in the second month and decreased over time. The study found no association between UTIs and acute rejection or between UTIs and post-transplant renal function. It concluded that UTIs may have little or no adverse impact on long-term graft survival outcomes. However, UTIs and their first recurrence were associated with increased hospitalization rates after transplantation, at 49% and 32% respectively. UTIs were most commonly seen in the first six months post-transplant, and the risk increased with factors such as older age, female gender, neurogenic bladder, and receiving the transplant abroad [9].

Multiple studies have been conducted to assess the impact of urinary tract infections (UTIs) following kidney transplantation. However, limited data is available on this subject in Saudi Arabia. In this study, our aim was to assess the impact of post-transplant UTIs on a cohort of renal transplant recipients who were followed and treated at King Abdulaziz Medical City (KAMC) from 2016 to 2022. Additionally, we sought to investigate potential recipient-related risk factors for the development of UTIs, identify the most frequently occurring pathogens causing UTIs, and examine the rate of acute rejection incidents following kidney transplantation in relation to UTIs. Finally, we aimed to identify the most common causes of end-stage renal disease (ESRD) that led to kidney transplantation in this patient group.

## Materials and methods

Study population and data selection 

Our hospital information system was retrospectively reviewed for all kidney transplant patients at the surgery department at King Abdulaziz Medical Center in Riyadh, Saudi Arabia. All adult patients (age ≥ 18 years) with early UTI who underwent KT at our center between 2016 and 2022 were included in the final analysis. Young patients (age ≤ 18 years) were excluded. A list of patients who had a kidney transplant during this period was obtained from the Patient Information Department. Medical records were reviewed for demographic data, including patient’s gender, age, time on dialysis, and cause of end-stage renal disease; such as diabetic nephropathy, glomerulonephritis, hypertensive nephrosclerosis, polycystic kidney disease, chronic interstitial nephritis, lupus nephritis, and IgA nephropathy. Information on previous transplantations, type of transplantation, pre- and post-transplant diabetes mellitus, postoperative antimicrobial therapy, and immunosuppressive regimen was also reviewed. We identified patients who had ureteral double-J stents implanted during the transplant and recorded the stent duration for them. Serum creatinine and estimated glomerular filtration rate (eGFR) were recorded after one month of transplantation.

Urinary tract infection 

UTI was classified into four different categories: asymptomatic bacteriuria, with no associated symptoms of UTI and no systemic clinical symptoms; uncomplicated acute simple cystitis, associated with local urinary symptoms; complicated UTI, associated with systemic manifestations; and recurrent UTI. A systematic review of each UTI episode was conducted, including time to onset after transplantation, type of infection, causative agent, microbiological diagnosis, treatment regimens, and duration of antibiotic treatment.

Immunosuppressive treatment 

Depending on the patient’s immunological risk, one of three induction therapies was used: none, basiliximab, or antithymocyte globulin (ATG). Postoperatively, patients were treated with tacrolimus, mycophenolate sodium, and prednisolone. Additionally, based on their follow-up presentation, they could be given cyclosporine, mammalian target of rapamycin (mTOR) inhibitors (such as sirolimus and everolimus), and mycophenolate mofetil.

Statistical analysis

Statistical analysis was performed using RStudio (R version 4.3.0). We used frequencies and percentages to express categorical variables, whereas numerical variables were expressed as median and interquartile ranges (IQRs). Statistical differences between patients with and without UTI were assessed using Pearson's chi-squared test or Fisher's exact test for categorical variables where applicable. The differences in terms of the continuous variables were explored using a Wilcoxon rank sum test. Statistical significance was considered at p < 0.05.

## Results

Demographic and clinical characteristics

**Figure 1:**
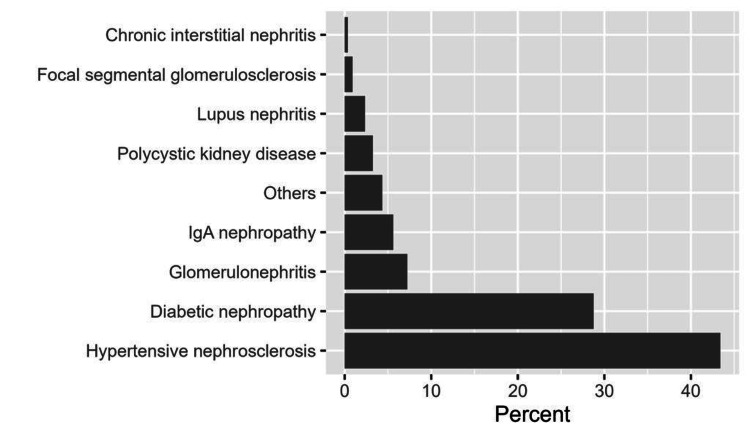
The proportion of ESRD causes among patients under study. ESRD: end-stage renal disease.

Initially, we collected data on 583 patients. However, we excluded eight records of patients aged <18 years and 22 records of those who had an infection before transplantation. Therefore, a total of 553 records were ultimately included in the analysis. More than half of the patients were males (62.4%), and 31.1% of them were aged 45 to <60 years. The median (IQR) BMI was 27.2 kg/m^2^ (22.8, 31.2). The majority of patients (89.7%) were on dialysis with a cumulative time of 18.0 months (9.0 to 36.0) on dialysis (Table 1). Of the 532 patients, the most commonly reported causes of end-stage renal disease (ESRD) were hypertensive nephrosclerosis (43.4%) and diabetic nephropathy (28.8%, Figure 1). Pre-existing urinary tract abnormalities were prevalent among 6.5% of patients, and 6.9% of them had undergone a previous transplantation. More details about the demographic and clinical characteristics are provided in Table 1.

**Table 1:** Demographic and clinical characteristics of patients. BPH: benign prostatic hyperplasia.

Parameter	Category	Overall, N = 553	UTI		P-Value	Missing
			No, N = 323	Yes, N = 230		
Age	18 to <30	132 (23.9%)	77 (58.3%)	55 (41.7%)	0.303	0 (0%)
	30 to <45	153 (27.7%)	93 (60.8%)	60 (39.2%)		
	45 to <60	172 (31.1%)	105 (61.0%)	67 (39.0%)		
	60 or more	96 (17.4%)	48 (50.0%)	48 (50.0%)		
Recipient gender	Male	345 (62.4%)	265 (76.8%)	80 (23.2%)	<0.001	0 (0%)
	Female	208 (37.6%)	58 (27.9%)	150 (72.1%)		
Blood group						
A+	No	411 (74.3%)	246 (59.9%)	165 (40.1%)	0.241	0 (0%)
	Yes	142 (25.7%)	77 (54.2%)	65 (45.8%)		
A-	No	535 (96.7%)	309 (57.8%)	226 (42.2%)	0.090	0 (0%)
	Yes	18 (3.3%)	14 (77.8%)	4 (22.2%)		
B+	No	445 (80.5%)	258 (58.0%)	187 (42.0%)	0.676	0 (0%)
	Yes	108 (19.5%)	65 (60.2%)	43 (39.8%)		
B-	No	539 (97.5%)	315 (58.4%)	224 (41.6%)	0.922	0 (0%)
	Yes	14 (2.5%)	8 (57.1%)	6 (42.9%)		
O+	No	330 (59.7%)	195 (59.1%)	135 (40.9%)	0.692	0 (0%)
	Yes	223 (40.3%)	128 (57.4%)	95 (42.6%)		
O-	No	535 (96.7%)	310 (57.9%)	225 (42.1%)	0.227	0 (0%)
	Yes	18 (3.3%)	13 (72.2%)	5 (27.8%)		
AB+	No	532 (96.2%)	310 (58.3%)	222 (41.7%)	0.740	0 (0%)
	Yes	21 (3.8%)	13 (61.9%)	8 (38.1%)		
AB-	No	549 (99.3%)	320 (58.3%)	229 (41.7%)	0.645	0 (0%)
	Yes	4 (0.7%)	3 (75.0%)	1 (25.0%)		
Weight	kg	71.6 (60.5, 83.0)	74.0 (62.0, 84.0)	69.0 (58.0, 81.3)	0.008	39 (7.1%)
Height	cm	164.0 (157.0, 171.0)	167.0 (160.0, 172.0)	158.0 (153.3, 165.0)	<0.001	46 (8.3%)
BMI	kg/m^2^	27.2 (22.8, 31.2)	26.9 (22.6, 30.9)	27.8 (23.4, 31.7)	0.057	47 (8.5%)
BMI	Underweight	24 (4.7%)	12 (50.0%)	12 (50.0%)	0.313	47 (8.5%)
	Healthy	167 (33.0%)	107 (64.1%)	60 (35.9%)		
	Overweight	159 (31.4%)	90 (56.6%)	69 (43.4%)		
	Obese	156 (30.8%)	87 (55.8%)	69 (44.2%)		
Dialysis	No	39 (7.1%)	27 (69.2%)	12 (30.8%)	0.358	0 (0%)
	Yes	496 (89.7%)	286 (57.7%)	210 (42.3%)		
	Unknown	18 (3.3%)	10 (55.6%)	8 (44.4%)		
Cumulative time on dialysis (months)	months	18.0 (9.0, 36.0)	13.0 (8.0, 36.0)	24.0 (10.0, 48.0)	0.003	168 (30%)
Pre-existing urinary tract abnormality	Vesicoureteric reflux	14 (2.5%)	7 (50.0%)	7 (50.0%)	0.024	0 (0%)
	Bladder dysfunction	6 (1.1%)	0 (0.0%)	6 (100.0%)		
	BPH	14 (2.5%)	10 (71.4%)	4 (28.6%)		
	Hydronephrosis	2 (0.4%)	1 (50.0%)	1 (50.0%)		
	None	517 (93.5%)	305 (59.0%)	212 (41.0%)		
Previous transplantation	No	500 (90.4%)	293 (58.6%)	207 (41.4%)	0.918	0 (0%)
	Yes	38 (6.9%)	22 (57.9%)	16 (42.1%)		
	Unknown	15 (2.7%)	8 (53.3%)	7 (46.7%)		
Type of transplantation	Deceased-donor kidney transplant	86 (15.6%)	46 (53.5%)	40 (46.5%)	0.466	1 (0.2%)
	Living-donor kidney transplant	439 (79.5%)	263 (59.9%)	176 (40.1%)		
	Preemptive kidney transplant	16 (2.9%)	8 (50.0%)	8 (50.0%)		
	Unknown	11 (2.0%)	5 (45.5%)	6 (54.5%)		
Pre-posttransplant diabetes mellitus	No	334 (60.5%)	196 (58.7%)	138 (41.3%)	0.837	1 (0.2%)
	Yes	218 (39.5%)	126 (57.8%)	92 (42.2%)		
Posttransplant diabetes mellitus	No	302 (55.1%)	179 (59.3%)	123 (40.7%)	0.577	5 (0.9%)
	Yes	246 (44.9%)	140 (56.9%)	106 (43.1%)		

Post-transplantation characteristics

The laboratory parameters of patients under study one month after kidney transplantation are demonstrated in Table 2. Acute rejection occurred in 13.3% of patients. Immunosuppressive regimens were used in all patients for whom records were available. The most common immunosuppressive medications were tacrolimus (97.5%), prednisolone (96.4%), and mycophenolic acid (91.7%) (Table 2).

Factors associated with UTI

UTI was significantly more prevalent among females (72.1% vs. 23.2% among males, p < 0.001) and those with pre-existing bladder dysfunction (100.0%) compared to those with other pre-existing urinary tract abnormalities (p = 0.024). Additionally, patients with UTI had significantly longer cumulative periods on dialysis (median = 24.0 days, IQR = 10.0 to 48.0) compared to those without UTI (median = 13.0 days, IQR = 8.0 to 36.0, p = 0.003, Table 1). Concerning post-transplantation laboratory parameters, UTI was significantly associated with lower concentrations of serum creatinine (median = 91.0, IQR = 71.0 to 115.8) compared to those without UTI (median = 110.0, IQR = 88.0 to 131.8, p < 0.001) and albumin (median = 40.0, IQR = 37.0 to 42.0) compared to those without UTI (median = 41.0, IQR = 39.3 to 44.0, p < 0.001). mTOR inhibitors were less frequently used among patients with UTI compared to those who did not have the infection (22.6% vs 47.1%, p < 0.001, Table 2).

**Table 2:** Post-transplantation characteristics of patients under study.

Parameter	Category	Overall, N = 553	UTI		P-Value	Missing
			No, N = 323	Yes, N = 230		
Post-transplantation lab parameters (after one month)	Serum creatinine	102.0 (80.0, 127.0)	110.0 (88.0, 131.8)	91.0 (71.0, 115.8)	<0.001	1 (0.2%)
	Albumin	41.0 (39.0, 43.0)	41.0 (39.3, 44.0)	40.0 (37.0, 42.0)	<0.001	1 (0.2%)
	eGFR	68.0 (54.0, 84.0)	67.0 (54.0, 80.0)	69.0 (55.0, 85.8)	0.297	1 (0.2%)
Episodes of acute rejection	No	355 (78.9%)	191 (53.8%)	164 (46.2%)	0.875	103 (19%)
	Yes	60 (13.3%)	31 (51.7%)	29 (48.3%)		
	Unknown	35 (7.8%)	20 (57.1%)	15 (42.9%)		
Duration of a double-J ureteral stent	Days	25.0 (14.0, 42.0)	24.0 (15.0, 39.0)	27.0 (14.0, 49.0)	0.239	45 (8.1%)
Immunosuppressive regimen used	No	0 (0.0%)	0 (NA%)	0 (NA%)	>0.999	1 (0.2%)
	Yes	552 (100.0%)	322 (58.3%)	230 (41.7%)		
Tacrolimus (prograf)	No	14 (2.5%)	10 (71.4%)	4 (28.6%)	0.314	1 (0.2%)
	Yes	538 (97.5%)	312 (58.0%)	226 (42.0%)		
Cyclosporine	No	551 (99.8%)	321 (58.3%)	230 (41.7%)	>0.999	1 (0.2%)
	Yes	1 (0.2%)	1 (100.0%)	0 (0.0%)		
Prednisolone	No	20 (3.6%)	13 (65.0%)	7 (35.0%)	0.535	4 (0.7%)
	Yes	529 (96.4%)	307 (58.0%)	222 (42.0%)		
Antithymocyte globulin	No	291 (52.7%)	159 (54.6%)	132 (45.4%)	0.063	1 (0.2%)
	Yes	261 (47.3%)	163 (62.5%)	98 (37.5%)		
Basiliximab	No	414 (75.0%)	243 (58.7%)	171 (41.3%)	0.765	1 (0.2%)
	Yes	138 (25.0%)	79 (57.2%)	59 (42.8%)		
Mycophenolic acid (MMF)	No	46 (8.3%)	29 (63.0%)	17 (37.0%)	0.508	2 (0.4%)
	Yes	505 (91.7%)	293 (58.0%)	212 (42.0%)		
mTOR inhibitor	No	425 (77.4%)	225 (52.9%)	200 (47.1%)	<0.001	4 (0.7%)
	Yes	124 (22.6%)	96 (77.4%)	28 (22.6%)		

Characteristics of urinary tract infections

**Figure 2:**
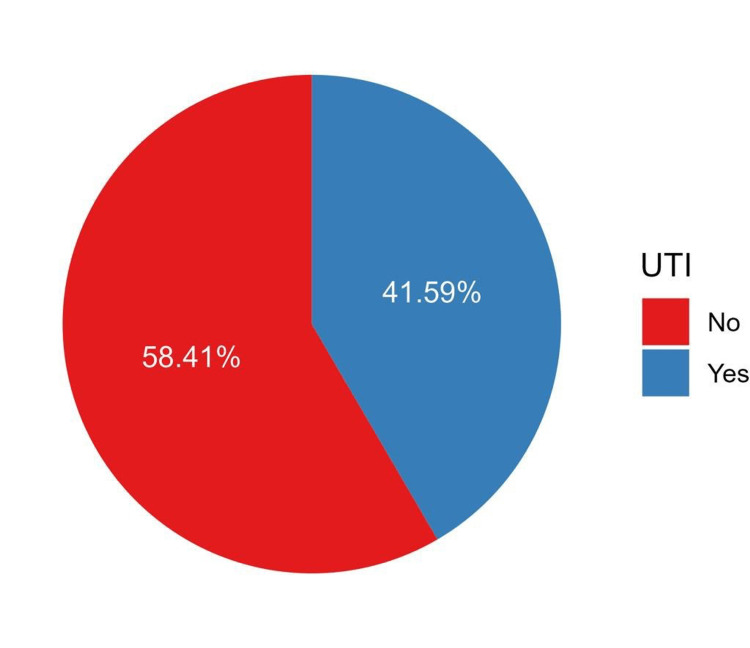
The proportion of UTI categories among patients under study. UTI: urinary tract infection.

A total of 230 patients had UTI in the current study, which represents 41.59% of the sample (Figure 2). The median (IQR) time from UTI incidence to transplantation was 44.5 days (IQR = 14.0 to 259.5). Asymptomatic bacteriuria was reported among 37.8%, and recurrent UTI was prevalent among 28.4% of patients. The most commonly isolated causative agents were *Escherichia coli *(51.0%) and *Klebsiella pneumoniae *(21.8%). Antibiotics were used among 86.5% of patients, of whom trimethoprim/sulfamethoxazole (65.8%) and ciprofloxacin (54.8%) were the most frequently prescribed antibiotics (Table 3).

**Table 3:** Characteristics of urinary tract infections and the used antibiotics.

Parameter	Category	N (%)	Missing
Type of urinary tract infection	Asymptomatic bacteriuria	84 (37.8%)	8 (3.5%)
	Acute simple cystitis	48 (21.6%)	
	Acute pyelonephritis/complicated UTI	27 (12.2%)	
	Recurrent UTI	63 (28.4%)	
Causative agents	Escherichia coli	105 (51.0%)	24 (10%)
	Klebsiella pneumoniae	45 (21.8%)	
	Enterococcus faecalis	19 (9.2%)	
	Pseudomonas aeruginosa	7 (3.4%)	
	Proteus mirabilis	0 (0.0%)	
	Staphylococcus epidermidis	1 (0.5%)	
	Candida spp	2 (1.0%)	
	Others	27 (13.1%)	
Time from UTI to transplant	Days	44.5 (14.0, 259.5)	
Antibiotics use	Yes	199 (86.5%)	0 (0%)
Type of antibiotic	Penicillins	81 (40.7%)	
	Cephalosporins	75 (37.7%)	
	Fosfomycin	3 (1.5%)	
	Ciprofloxacin	109 (54.8%)	
	Nitrofurantoin	69 (34.7%)	
	Carbapenems	76 (38.2%)	
	Fluoroquinolones	7 (3.5%)	
	Bactrim	131 (65.8%)	

## Discussion

UTIs have been reported to be significantly associated with an increased risk for acute graft rejection in the early post-transplant period. These infections can progress to severe conditions such as acute pyelonephritis, bacteremia, and urosepsis, all of which become independent risk factors affecting short- and long-term graft and patient survival [10]. In our study, 553 records were examined, revealing that over half of the patients were males (62.4%) and 31.1% were aged between 45 and 60 years.

Analysis of the dataset revealed that 230 (41.59%) of the patients experienced a UTI post-transplant. Of these, asymptomatic bacteriuria (AB) was reported in 37.8% and recurrent UTIs in 28.4%. Despite a recent decline in the incidence of post-transplant UTIs, the numbers remain high [11]. This suggests that medical staff may benefit from further training in the early prevention and treatment of UTIs post-kidney transplantation.

In terms of the primary causes of end-stage renal disease (ESRD) in our patient population, the most common were hypertensive nephrosclerosis (43.4%) and diabetic nephropathy (28.8%), findings that align with other studies [12,13]. When looking at the causative pathogens of UTI, *Escherichia coli* was the most common, accounting for 51% of cases, followed by *Klebsiella pneumoniae* at 21.8%. These findings corroborate a previous study where *Escherichia coli* and *Klebsiella pneumoniae* were identified in 82% of UTI cases among renal transplant patients [14].

Concerning treatment modalities, antibiotics were prescribed for 86.5% of the UTI cases in our study. The most commonly used antibiotics were trimethoprim/sulfamethoxazole (65.8%) and ciprofloxacin (54.8%). Multiple studies have shown the efficacy of trimethoprim/sulfamethoxazole as a prophylactic measure to reduce UTI incidence when compared to a placebo [15,16]. For treating uncomplicated UTIs, the "first-line" antibiotics recommended are amoxicillin, cephalexin, or trimethoprim-sulfamethoxazole. In cases of complicated or resistant infections, the use of potentiated β-lactams, such as amoxicillin-clavulanic acid, is recommended [17].

Our study corroborates previous findings by highlighting the significant association between UTIs and female gender (p < 0.001) as well as pre-existing bladder dysfunction (p = 0.024). Prior research has already identified female gender, older age, and the use of catheterization instruments as authentic risk factors for UTIs in both the general population and kidney transplant recipients [18,19,20].

In the context of kidney transplantation, specific factors like ureteral stent placement have been linked to a heightened risk of UTI [21,22]. Additionally, receiving a graft from a deceased donor and experiencing delayed graft function have also been identified as risk factors [19,20].

The role of immunosuppressive therapy in influencing UTI incidence cannot be overstated. Induction therapy has been associated with UTIs in some previous studies when analyzed univariately [23]. In another study involving 500 kidney transplant recipients followed over an average of 42 months, significant risk factors for UTIs were identified as older age, female gender, reflux kidney disease, use of azathioprine, and deceased donor as the source of the kidney graft.

Several factors might contribute to the elevated risk of UTIs in females. First, the anatomical proximity of the female urethra to the anus increases susceptibility to intestinal flora like E. coli, thus making retrograde infections more likely [13]. Second, differences between male and female urine proteomic patterns could influence urinary bacterial proliferation and inflammatory responses [24]. Lastly, post-menopausal women may experience a change in the acidic environment of the vagina due to reduced estrogen levels, thereby compromising natural infection-protection mechanisms [25].

Regarding the impact of UTI on graft survival, a previous study conducted among 601 kidney transplant patients revealed that recurrent UTI episodes increased the risk of graft loss by more than 2.5-fold [26]. In contrast, another study reported that UTI had no negative impact on short-term graft survival, findings that are similar to our own [9]. These differences could be attributed to variability in study methodology, the target population, or the data collection instruments used. Ultimately, the extent of UTI's impact on graft survival remains uncertain.

Since the data for this study were collected from a single center within a specific setting, they may represent a highly selected group of cases. Therefore, it may be difficult to generalize these findings to the broader community of kidney recipients.

## Conclusions

In conclusion, our study comprised 553 adult kidney transplant recipients. Our results indicated that nearly half of these recipients experienced a UTI, with *Escherichia coli *and *Klebsiella pneumoniae *as the most common causative organisms. Most patients were prescribed antibiotics as part of their treatment plan, with trimethoprim/sulfamethoxazole and ciprofloxacin being the most frequently prescribed options. A heightened risk for contracting a UTI was observed particularly among females and those with pre-existing urinary tract abnormalities. Importantly, our study found that UTIs had no negative impact on short-term graft survival. We recommend future studies to explore other potential variables affecting graft function in kidney transplant recipients. Additionally, we suggest monitoring the risk of UTI in patients who undergo ureteral stent placement after kidney transplantation, as existing literature indicates a possible correlation.
